# Synthesis, crystal structure and Hirshfeld surface analysis of 1,7-dimethyl-5a,6,11a,12-tetra­hydro­benzo[*b*]benzo[5,6][1,4]oxazino[2,3-*e*][1,4]oxazine

**DOI:** 10.1107/S2056989020010646

**Published:** 2020-08-18

**Authors:** Emine Berrin Çınar, Semanur Yeşilbağ, Onur Erman Doğan, Erbil Ağar, Necmi Dege, Eiad Saif

**Affiliations:** a Department of Physics, Faculty of Arts and Sciences, Ondokuz Mayıs University, Samsun, 55200, Turkey; b Department of Chemistry, Faculty of Arts and Sciences, Ondokuz Mayıs, University, Samsun, 55200, Turkey; c Department of Computer and Electronic Engineering Technology, Sana’a Community, College, Sana’a, Yemen

**Keywords:** crystal structure, Hirshfeld surface, DFT, oxazines

## Abstract

Mol­ecules of the title compound, C_16_H_16_N_2_O_2_, occupy special positions on the twofold rotation axes. The heterocyclic ring adopts a slightly twisted envelope conformation with one of the two junction carbon atoms as the flap. The mean planes through the two halves of the mol­ecule form a dihedral angle of 72.01 (2)°. In the crystal, mol­ecules are linked by pairs of C—H⋯O and N—H⋯C contacts into layers parallel to (100). H⋯H contacts make the largest contribution to the Hirshfeld surface (58.9%).

## Abstract 2

In the crystal, mol­ecules are linked by pairs of C—H⋯O and N—H⋯C contacts into layers parallel to (100). H⋯H contacts make the largest contribution to the Hirshfeld surface (58.9%).

## Chemical context   

The title oxazine derivative contains two six-membered heterocyclic rings located between two benzene rings. Oxazine-derived compounds are used in the synthesis of detergents, corrosion inhibitors and industrial dyes (Adib *et al.*, 2006 ▸). This class of mol­ecules has been studied extensively as they exhibit anti­tumor (Sriharsha *et al.*, 2006 ▸), anti­bacterial and anti­fungal (Belz *et al.*, 2013 ▸) activity. Oxazinooxazines are important heterocyclic precursors in the construction of heteropropellanes with applications in material sciences and medicinal chemistry (Dilmaç *et al.*, 2017 ▸). Such heterocycles can be synthesized by several methods (Konstanti­nova *et al.*, 2020 ▸), with the most direct route being the condensation of amino alcohols with either aldehydes or ketones (Hajji *et al.*, 2003 ▸). As the amino and hy­droxy groups are adjacent, 2-amino­phenol readily forms heterocycles. An inter­esting feature of the reaction is the stereo-selective transformation of glyoxal. We report herein the crystal structure and Hirshfeld surface analysis for a new oxazine derivative, 1,7-dimethyl-5a,6,11a,12-tetra­hydro­benzo[*b*]benzo[5,6][1,4]oxazino[2,3-*e*][1,4]oxazine.
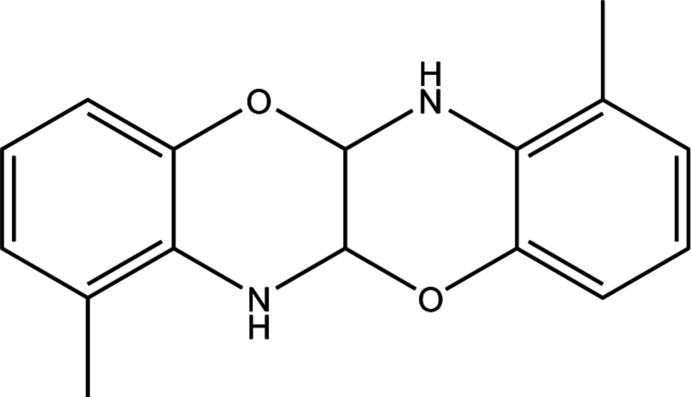


## Structural commentary   

**Figure 1:**
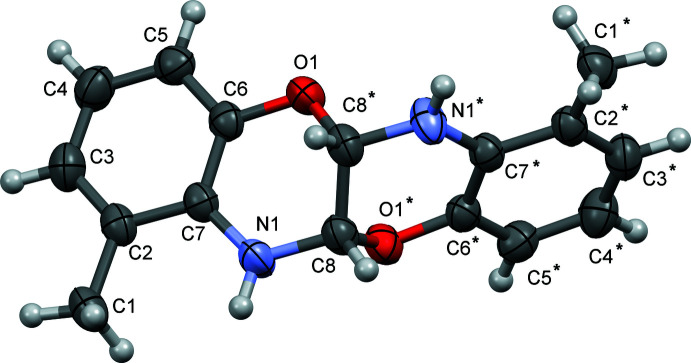
The mol­ecular structure of the title compound, showing the atom labelling and displacement ellipsoids drawn at the 40% probability level. Starred atoms are generated by the symmetry operation −*x* − 1, *y*, −*z* − 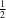.

The mol­ecular structure of the title compound (I) is shown in Fig. 1 ▸. The mol­ecules occupy special positions on the twofold rotation axes. The heterocyclic ring adopts a slightly twisted envelope conformation with the C8* [symmetry code: (*) −*x* − 1, *y*, −*z* − 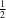] atom as the flap. Except for this atom, the symmetry-independent part of the mol­ecule (C2–C8/O1/N1) is nearly planar, the largest separation from the mean plane being 0.1267 (10) Å for O1. The mean planes of the two halves of the mol­ecule form a dihedral angle of 72.01 (2)°.

## Supra­molecular features   

**Figure 2:**
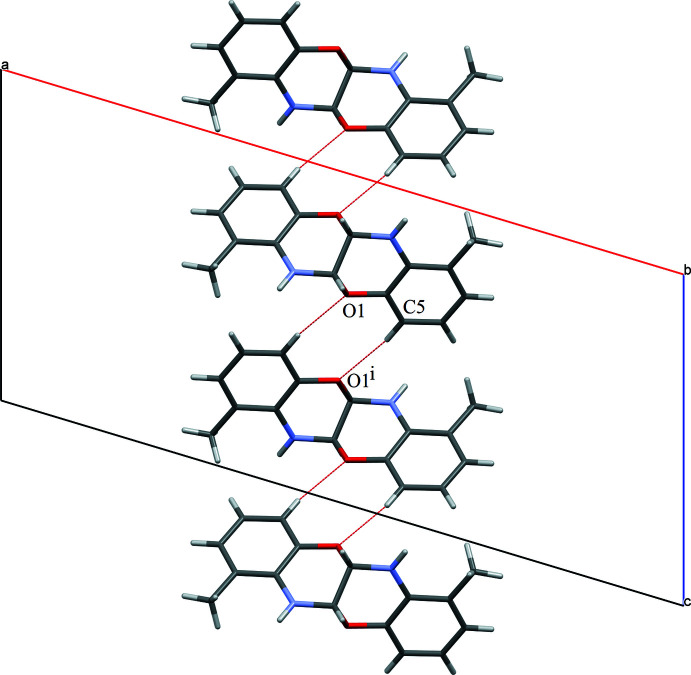
Chains of the title mol­ecules linked by pairs of C—H⋯O inter­actions.

**Figure 3:**
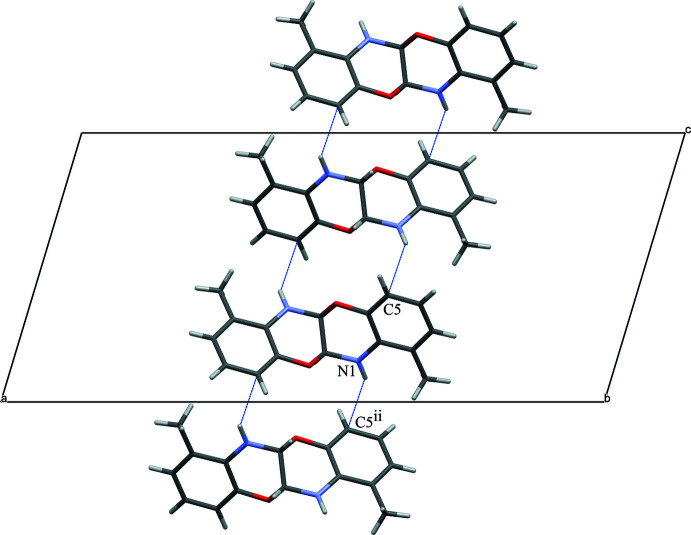
Chains of mol­ecules linked by pairs of N—H⋯C inter­actions.

**Figure 4:**
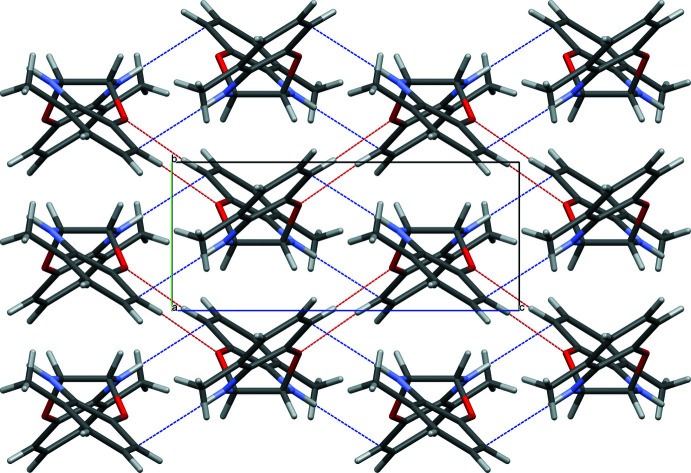
Layer of the title mol­ecules linked by C—H⋯O (red) and N—H⋯C (blue) inter­actions.

Surprisingly, no inter­molecular N—H⋯O contacts are observed in the title structure. Instead, C—H⋯O and N—H⋯C contacts are formed, the latter really being of the N—H⋯π type. Pairs of C—H⋯O contacts link the mol­ecules into zigzag chains along [001] (Table 1 ▸, Fig. 2 ▸). Pairs of N—H⋯O contacts also form zigzag chains of mol­ecules along [001] (Table 1 ▸, Fig. 3 ▸). As a result, layers parallel to (100) are formed (Fig. 4 ▸).

## Hirshfeld surface   

**Figure 5:**
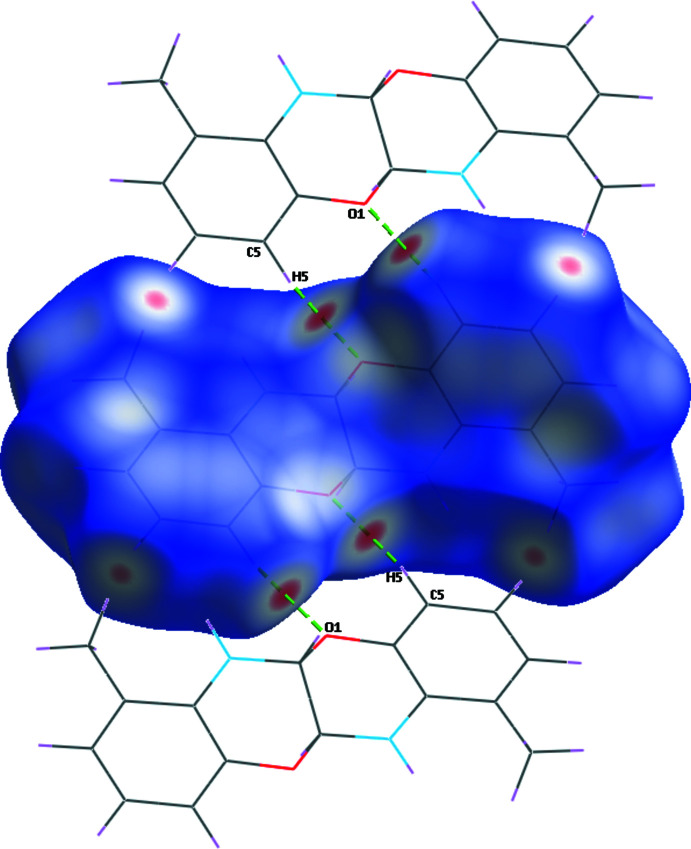
View of the three-dimensional Hirshfeld surface for the title mol­ecule plotted over *d*_norm_.

**Figure 6:**
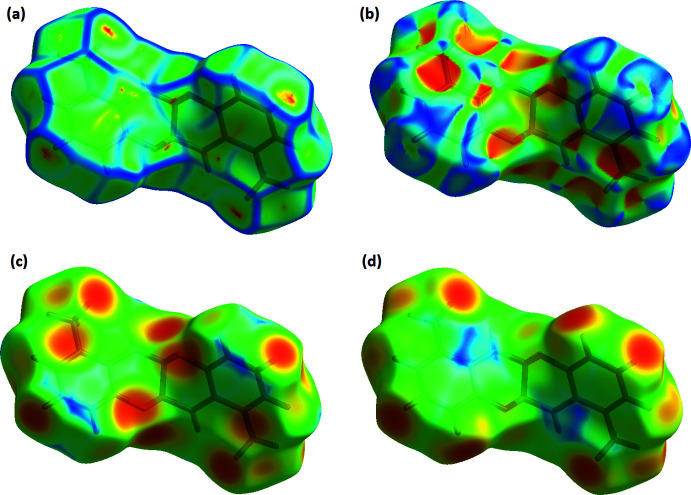
The Hirshfeld surfaces of the title mol­ecule mapped over (*a*) curvedness, (*b*) shape-index, (*c*) *d*_i_ and (*d*) *d*_e_.

**Figure 7:**
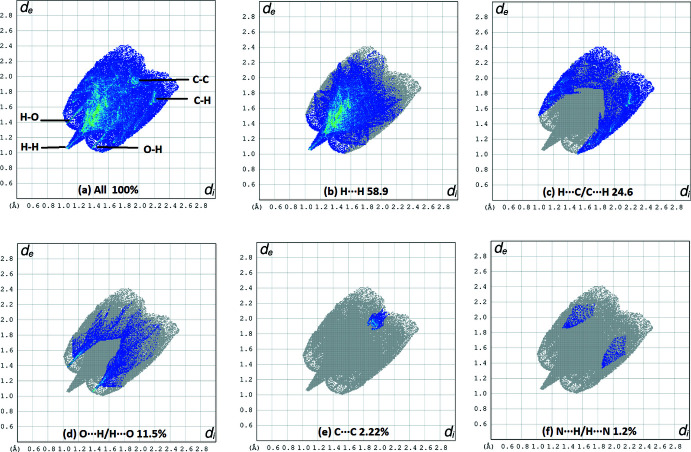
Two-dimensional fingerprint plot for the title mol­ecule (*a*) and those delineated into the specific types of inter­actions (*b–f*).

The Hirshfeld surfaces were generated using *Crystal Explorer 17.5* (Turner *et al.*, 2017 ▸). The *d*_norm_ mapping was performed in the range of −0.186 to 1.019 arbitrary units. Red spots on the *d*_norm_ surface (Fig. 5 ▸) indicate regions of C—H⋯O inter­actions. However, the N—H⋯C contacts do not cause red spots on the Hirshfeld surface. Other red spots are due to the H⋯H inter­actions, as can be understood from the fingerprint plot. The characteristic flat surface patches caused by planar stacking are shown in Fig. 6 ▸ *a*. The shape-index map (Fig. 6 ▸ *b*) does not contain red and blue triangles related to π–π inter­actions. Fig. 6 ▸ *c*,*d* show the *d*_i_ and *d*_e_ surfaces, respectively. Fig. 7 ▸ presents the two-dimensional fingerprint plot for the title mol­ecule and those delineated into the specific types of inter­actions. The H⋯H contacts make the largest contribution to the Hirshfeld surface (58.9%). The H⋯C/C⋯H inter­actions are seen at the edges of two-dimensional fingerprint drawings, with a general contribution of 24.6%.

## Database survey   

A search of the Cambridge Structural Database (CSD, version 5.40, update of August 2019; Groom *et al.*, 2016 ▸) using 1-benzyl-3,4-di­hydro­quinoxalin-2(1*H*)-one as the main skeleton revealed the presence of four structures similar to the title compound. These are 2,8-di-*t*-butyl-5a,6,11a,12-tetra­hydro­[1,4]benzoxazino[3,672-*b*][1,4]benzoxazine (MOYJOC; Niklas *et al.*, 2019 ▸), 5a,6,11a,12-tetra­hydro­[1,4]benz­oxa­zino[3,2-*b*][1,4]benzoxazine (FIGVOG; Tauer *et al.*, 1986 ▸), 5a,6,11a,12-tetra­hydro-5a,11a-dimethyl-1,4-benzoxazino[3,2-*b*][1,4]benzoxazine (ABEQAA; Hai-Yan *et al.*, 2004 ▸) and *N*,*N*′-di-5a,6,11a,12-tetra­hydro­[1,4]benzoxazino[3,2]benzoxazine (BAJNIJ; Farfán *et al.*, 1992 ▸). In the structures MOYJOC and FIGVOG, the dihedral angles between the two approximately planar halves of the mol­ecule [67.11 (3) and 64.28 (2)°, respectively] are smaller than in (I). In MOYJOC, both NH groups are involved in hydrogen bonds with the heterocyclic oxygen atoms. In FIGVOG, only one NH group takes part in such hydrogen bonding, while the other makes an N—H⋯C contact similar to that observed in (I). In ABEQAA, the hydrogen atoms at the bridge C atoms (C8 and C8* in the title mol­ecule) are replaced by methyl groups. As a result, the dihedral angle increases to 81.70 (2)°. In this structure, both NH groups form weak inter­molecular N—H⋯O hydrogen bonds.

## Synthesis and crystallization   

To a solution of 2-amino-3-methyl­phenol (21.8 mg, 0.177 mmol) in ethanol (20 ml), was added glyoxal (40 wt % solution in H_2_O) (12.8 mg, 0.089 mmol) dissolved in ethanol (20 ml) and the mixture was refluxed for 12 h. The orange product obtained was washed with ether and recrystallized from ethanol at room temperature (m.p. 472-475 K, yield 67%).
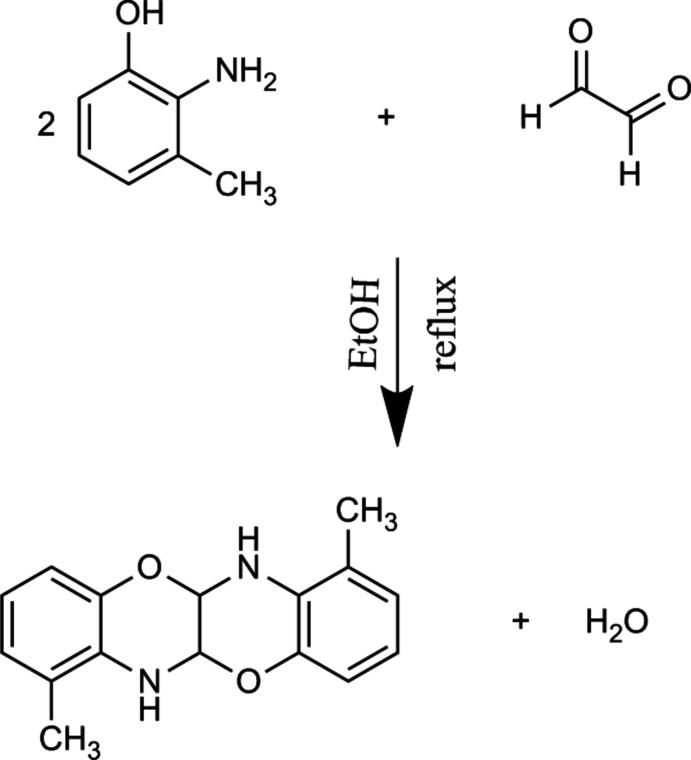


## Refinement   

Crystal data, data collection and structure refinement details are summarized in Table 2 ▸. All hydrogen atoms were constrained to ride on their parent atoms with C—H = 0.93, 0.96 and 0.98 Å for aromatic, methyl and methine H atoms, respectively, and with N—H = 0.86 Å. Isotropic displacement parameters of these atoms were constrained to 1.5*U*_eq_(C) for the methyl group and to 1.2*U*_eq_(C,N) for all other H atoms.

## Supplementary Material

Crystal structure: contains datablock(s) I. DOI: 10.1107/S2056989020010646/yk2135sup1.cif

Structure factors: contains datablock(s) I. DOI: 10.1107/S2056989020010646/yk2135Isup4.hkl

Supporting information file. DOI: 10.1107/S2056989020010646/yk2135Isup3.cml

CCDC reference: 2021045

Additional supporting information:  crystallographic information; 3D view; checkCIF report

## Figures and Tables

**Table 1:** Hydrogen-bond geometry (Å, °)

*D*—H⋯*A*	*D*—H	H⋯*A*	*D*⋯*A*	*D*—H⋯*A*
C5—H5⋯O1^i^	0.93	2.59	3.513 (2)	172
N1—H1⋯C5^ii^	0.86	2.64	3.375 (2)	144

Symmetry codes: (i) 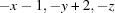; (ii) 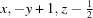.

**Table 2:** Experimental details

Crystal data	
Chemical formula	C_16_H_16_N_2_O_2_
*M*_r_	268.31
Crystal system, space group	Monoclinic, *C*2/*c*
Temperature (K)	296
*a*, *b*, *c* (Å)	24.798 (3), 4.7133 (4), 11.5330 (14)
β (°)	106.751 (9)
*V* (Å^3^)	1290.8 (3)
*Z*	4
Radiation type	Mo *K*α
μ (mm^−1^)	0.09
Crystal size (mm)	0.78 × 0.42 × 0.13

Data collection	
Diffractometer	Stoe IPDS 2
Absorption correction	Integration (*X-RED32*; Stoe & Cie, 2002 ▸)
*T*_min_, *T*_max_	0.941, 0.989
No. of measured, independent and observed [*I* > 2σ(*I*)] reflections	5580, 2194, 1024
*R*_int_	0.059
(sin θ/λ)_max_ (Å^−1^)	0.745

Refinement	
*R*[*F* ^2^ > 2σ(*F* ^2^)], *wR*(*F* ^2^), *S*	0.049, 0.134, 0.88
No. of reflections	2194
No. of parameters	92
H-atom treatment	H-atom parameters constrained
Δρ_max_, Δρ_min_ (e Å^−3^)	0.15, −0.16

Computer programs: *X-AREA* and *X-RED32* (Stoe & Cie, 2002 ▸), *SHELXT2018/3* (Sheldrick, 2015*a* ▸), *SHELXL2018/3* (Sheldrick, 2015*b* ▸), *OLEX2* (Dolomanov *et al.*, 2009 ▸), *Mercury* (Macrae *et al.*, 2020 ▸), *WinGX* (Farrugia, 2012 ▸), *PLATON* (Spek, 2020 ▸) and *publCIF* (Westrip, 2010 ▸).

## supplementary crystallographic information

### Crystal data

**Table:**

C_16_H_16_N_2_O_2_	*D*_x_ = 1.381 Mg m^−^^3^
*M**_r_* = 268.31	Melting point = 472–475 K
Monoclinic, *C*2/*c*	Mo *K*α radiation, λ = 0.71073 Å
*a* = 24.798 (3) Å	Cell parameters from 3858 reflections
*b* = 4.7133 (4) Å	θ = 1.8–32.0°
*c* = 11.5330 (14) Å	µ = 0.09 mm^−^^1^
β = 106.751 (9)°	*T* = 296 K
*V* = 1290.8 (3) Å^3^	Plate, orange
*Z* = 4	0.78 × 0.42 × 0.13 mm
*F*(000) = 568	

### Data collection

**Table:**

Stoe IPDS 2 diffractometer	2194 independent reflections
Radiation source: sealed X-ray tube, 12 x 0.4 mm long-fine focus'	1024 reflections with *I* > 2σ(*I*)
Plane graphite monochromator	*R*_int_ = 0.059
Detector resolution: 6.67 pixels mm^-1^	θ_max_ = 32.0°, θ_min_ = 3.4°
rotation method scans	*h* = −35→36
Absorption correction: integration (X-RED32; Stoe & Cie, 2002)	*k* = −7→6
*T*_min_ = 0.941, *T*_max_ = 0.989	*l* = −16→16
5580 measured reflections	

### Refinement

**Table:**

Refinement on *F*^2^	Primary atom site location: structure-invariant direct methods
Least-squares matrix: full	Secondary atom site location: difference Fourier map
*R*[*F*^2^ > 2σ(*F*^2^)] = 0.049	Hydrogen site location: inferred from neighbouring sites
*wR*(*F*^2^) = 0.134	H-atom parameters constrained
*S* = 0.88	*w* = 1/[σ^2^(*F*_o_^2^) + (0.0639*P*)^2^] where *P* = (*F*_o_^2^ + 2*F*_c_^2^)/3
2194 reflections	(Δ/σ)_max_ < 0.001
92 parameters	Δρ_max_ = 0.15 e Å^−^^3^
0 restraints	Δρ_min_ = −0.16 e Å^−^^3^

### Special details

**Table:**

Geometry. All esds (except the esd in the dihedral angle between two l.s. planes) are estimated using the full covariance matrix. The cell esds are taken into account individually in the estimation of esds in distances, angles and torsion angles; correlations between esds in cell parameters are only used when they are defined by crystal symmetry. An approximate (isotropic) treatment of cell esds is used for estimating esds involving l.s. planes.

### Fractional atomic coordinates and isotropic or equivalent isotropic displacement parameters (Å^2^)

**Table:**

	*x*	*y*	*z*	*U*_iso_*/*U*_eq_	
O1	−0.50672 (4)	0.7122 (2)	−0.13429 (9)	0.0582 (3)	
N1	−0.57174 (5)	0.4629 (3)	−0.34733 (13)	0.0622 (4)	
H1	−0.591648	0.369876	−0.409012	0.075*	
C7	−0.59760 (6)	0.6173 (3)	−0.27650 (13)	0.0484 (3)	
C2	−0.65643 (6)	0.6435 (3)	−0.30692 (14)	0.0531 (4)	
C6	−0.56462 (6)	0.7472 (3)	−0.17100 (13)	0.0497 (3)	
C8	−0.51247 (6)	0.4590 (3)	−0.31772 (14)	0.0545 (4)	
H8	−0.500090	0.289696	−0.352275	0.065*	
C3	−0.67936 (7)	0.8104 (4)	−0.23575 (17)	0.0657 (5)	
H3	−0.718296	0.828025	−0.255126	0.079*	
C5	−0.58834 (7)	0.9190 (3)	−0.10285 (15)	0.0599 (4)	
H5	−0.565754	1.011706	−0.034970	0.072*	
C1	−0.69244 (7)	0.4830 (4)	−0.41332 (17)	0.0675 (5)	
H1A	−0.682704	0.536853	−0.485013	0.101*	
H1B	−0.731357	0.526107	−0.423392	0.101*	
H1C	−0.686334	0.283070	−0.399720	0.101*	
C4	−0.64605 (8)	0.9525 (4)	−0.13620 (18)	0.0709 (5)	
H4	−0.662501	1.070905	−0.091513	0.085*	

### Atomic displacement parameters (Å^2^)

**Table:**

	*U*^11^	*U*^22^	*U*^33^	*U*^12^	*U*^13^	*U*^23^
O1	0.0456 (6)	0.0679 (7)	0.0562 (6)	−0.0052 (4)	0.0068 (5)	−0.0074 (5)
N1	0.0411 (7)	0.0735 (8)	0.0664 (8)	−0.0036 (6)	0.0067 (6)	−0.0223 (7)
C7	0.0436 (7)	0.0457 (7)	0.0545 (8)	−0.0027 (6)	0.0122 (6)	0.0014 (6)
C2	0.0435 (7)	0.0526 (8)	0.0612 (9)	−0.0031 (6)	0.0120 (7)	0.0087 (7)
C6	0.0449 (7)	0.0509 (7)	0.0534 (8)	−0.0046 (6)	0.0146 (6)	0.0049 (7)
C8	0.0429 (7)	0.0554 (8)	0.0621 (9)	0.0023 (6)	0.0101 (7)	−0.0047 (7)
C3	0.0487 (9)	0.0707 (10)	0.0806 (12)	0.0037 (8)	0.0234 (9)	0.0044 (9)
C5	0.0648 (10)	0.0610 (9)	0.0565 (9)	−0.0076 (7)	0.0214 (7)	−0.0040 (7)
C1	0.0450 (8)	0.0771 (10)	0.0726 (11)	−0.0070 (7)	0.0044 (7)	0.0036 (9)
C4	0.0669 (11)	0.0733 (11)	0.0811 (12)	0.0031 (8)	0.0349 (10)	−0.0104 (10)

### Geometric parameters (Å, º)

**Table:**

O1—C6	1.3846 (17)	C8—C8^i^	1.505 (3)
O1—C8^i^	1.4521 (18)	C8—H8	0.9800
N1—C7	1.3822 (19)	C3—C4	1.379 (3)
N1—C8	1.4099 (19)	C3—H3	0.9300
N1—H1	0.8600	C5—C4	1.380 (2)
C7—C6	1.397 (2)	C5—H5	0.9300
C7—C2	1.4041 (19)	C1—H1A	0.9600
C2—C3	1.373 (2)	C1—H1B	0.9600
C2—C1	1.498 (2)	C1—H1C	0.9600
C6—C5	1.373 (2)	C4—H4	0.9300
			
C6—O1—C8^i^	113.95 (11)	O1^i^—C8—H8	109.8
C7—N1—C8	119.51 (12)	C8^i^—C8—H8	109.8
C7—N1—H1	120.2	C2—C3—C4	121.60 (15)
C8—N1—H1	120.2	C2—C3—H3	119.2
N1—C7—C6	119.38 (12)	C4—C3—H3	119.2
N1—C7—C2	121.63 (14)	C6—C5—C4	119.27 (16)
C6—C7—C2	118.98 (14)	C6—C5—H5	120.4
C3—C2—C7	118.72 (15)	C4—C5—H5	120.4
C3—C2—C1	121.82 (14)	C2—C1—H1A	109.5
C7—C2—C1	119.43 (15)	C2—C1—H1B	109.5
C5—C6—O1	118.20 (13)	H1A—C1—H1B	109.5
C5—C6—C7	121.14 (13)	C2—C1—H1C	109.5
O1—C6—C7	120.64 (12)	H1A—C1—H1C	109.5
N1—C8—O1^i^	109.28 (12)	H1B—C1—H1C	109.5
N1—C8—C8^i^	109.82 (15)	C3—C4—C5	120.05 (16)
O1^i^—C8—C8^i^	108.29 (9)	C3—C4—H4	120.0
N1—C8—H8	109.8	C5—C4—H4	120.0
			
C8—N1—C7—C6	−5.4 (2)	N1—C7—C6—O1	−2.8 (2)
C8—N1—C7—C2	175.60 (14)	C2—C7—C6—O1	176.19 (13)
N1—C7—C2—C3	−177.10 (14)	C7—N1—C8—O1^i^	−82.21 (17)
C6—C7—C2—C3	3.9 (2)	C7—N1—C8—C8^i^	36.44 (15)
N1—C7—C2—C1	4.7 (2)	C7—C2—C3—C4	0.3 (2)
C6—C7—C2—C1	−174.29 (14)	C1—C2—C3—C4	178.44 (16)
C8^i^—O1—C6—C5	159.07 (13)	O1—C6—C5—C4	−178.67 (14)
C8^i^—O1—C6—C7	−22.75 (17)	C7—C6—C5—C4	3.2 (2)
N1—C7—C6—C5	175.29 (14)	C2—C3—C4—C5	−2.9 (3)
C2—C7—C6—C5	−5.7 (2)	C6—C5—C4—C3	1.1 (2)

Symmetry code: (i) −*x*−1, *y*, −*z*−1/2.

### Hydrogen-bond geometry (Å, º)

**Table:**

*D*—H···*A*	*D*—H	H···*A*	*D*···*A*	*D*—H···*A*
C5—H5···O1^ii^	0.93	2.59	3.513 (2)	172
N1—H1···C5^iii^	0.86	2.64	3.375 (2)	144

Symmetry codes: (ii) −*x*−1, −*y*+2, −*z*; (iii) *x*, −*y*+1, *z*−1/2.

### Selected bond lengths, bond and dihedral angles (° A, °) in the title structure

**Table:**

Parameters	Å, °
O1—C6	1.3846 (17)
O1—C8*	1.4521 (18)
N1—C8	1.4099 (19)
N1—C7	1.3822 (19)
C8—C8*	1.505 (3)
C6—C7	1.397 (2)
O1*—C8—C8*	108.29 (9)
N1—C8—C8*	109.82 (15)
N1—C8—O1*	109.28 (12)
C6—O1—C8*	113.95 (11)
C7—N1—C8—C8*	36.44 (15)
C8*—O1—C6—C7	-22.75 (17)
C2—C7—C6—C5	-5.7 (2)
N1—C7—C6—O1	-2.8 (2)
